# Multiple primary malignancies and prolonged survival in a patient with widespread metastatic cutaneous melanoma

**DOI:** 10.1097/CMR.0000000000000426

**Published:** 2018-01-17

**Authors:** Anna M. Rose, Utsav K. Radia, Rong Luo, Helen Kalirai, Channa N. Jayasena, Philip Luthert, Sarah E. Coupland, Geoffrey E. Rose

**Affiliations:** a UCL Institute of Ophthalmology, University College; b Department of Medicine, Imperial College; c Orbital Service, Moorfields Eye Hospital, London; d Department of Molecular and Clinical Cancer Medicine, Institute of Translational Medicine, University of Liverpool, Liverpool, UK

We report a possibly unique patient who developed four different primary malignancies, in the absence of any detectable germline genetic abnormality or a family history of malignancy. The patient has also survived for more than 15 years after local treatments (without immunotherapy) of cutaneous melanoma metastatic to her orbit, brain, skeleton and peritoneum. A 68-year-old White British woman was referred to the Orbital Service at Moorfields Eye Hospital with a 4-month history of right proptosis and true binocular diplopia, this being 41 months after having a 0.8-mm superficial spreading malignant melanoma of her left ankle treated with wide local resection. She underwent resection of a left frontal lobe metastasis a year before orbital presentation. Computed tomographic scan showed a well-defined Supero-temporal soft-tissue mass in the right retrobulbar fat (Fig. 1a and b). The patient underwent anterior orbitotomy through an upper eyelid skin-crease incision, with macroscopic excision of the mass, and histological examination showed infiltrative melanoma compatible with metastasis from her previous skin malignancy (Fig. 2). She underwent external beam radiotherapy (30 Gy in five fractions over 2 weeks) to the right retrobulbar tissues.

**Fig. 1:**
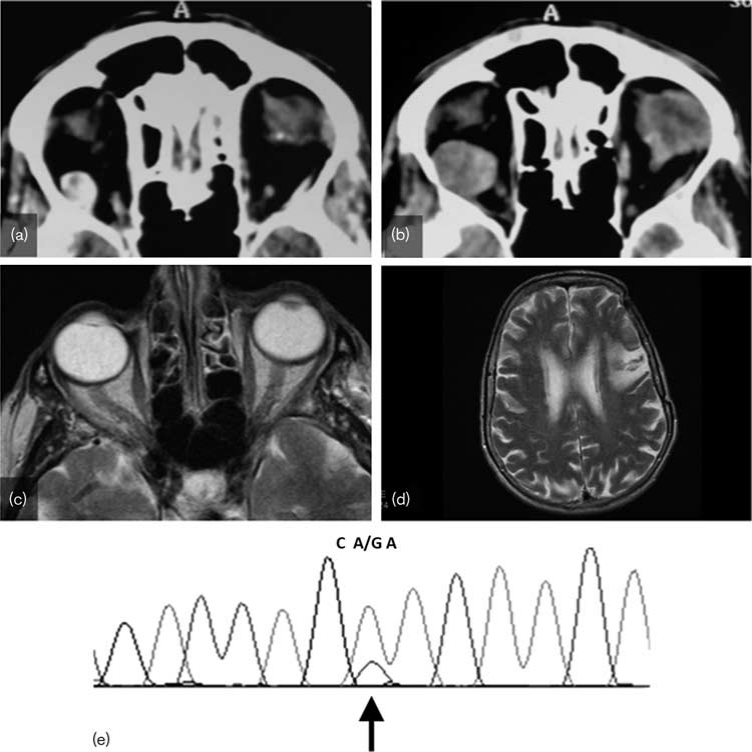
Imaging of orbital and intracranial disease in a patient with disseminated cutaneous malignant melanoma. (a, b) Orbital computed tomography at presentation showed a well-defined soft-tissue mass supero-temporally in the right retrobulbar fat. (c) MRI showed no recurrence of orbital disease 14 years after excision of the orbital mass and radiotherapy. (d) MRI showing gliosis at the site of previous surgical excision and radiotherapy for left frontal metastases, but without disease recurrence at 15 years after treatment of the intracranial disease. (e) Sequencing of DNA extracted from the patient’s metastatic melanoma excised from her orbit, this showing a heterozygous point mutation (c.A182G; p.Q61R) (arrowed) in exon 3 of *NRAS*.

**Fig. 2:**
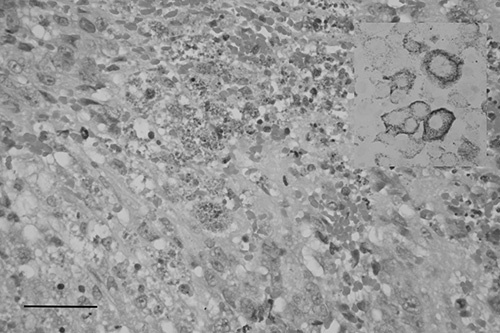
Light micrograph of resected orbital tumour showing a population of pleomorphic cells with large nuclei and prominent nucleoli (scale bar=40 µm). Pigmentation is present, consistent with metastatic cutaneous melanoma. The inset panel shows immunoreactivity for HMB45.

Three months after the orbitotomy (44 months after the primary skin tumour), the patient was found to have a right femoral bony metastasis, which responded well to local radiotherapy and insertion of an intramedullary femoral nail. At 57 months after the primary skin lesion, the patient underwent resection of multiple retroperitoneal deposits of metastatic melanoma. There has been no further recurrence of metastatic cutaneous melanoma at 17 years following removal of the primary tumour (14 years after orbital presentation) (Fig. 1c and d).

The patient also had a melanoma of soft parts resected from her left thigh musculature at the age of 35 years. At age 48, she underwent left lumpectomy, radiotherapy and axillary nodal clearance for breast adenocarcinoma. The patient more recently has developed – at age 80 – squamous cell carcinoma of her scalp, this being treated with wide local excision; the tumour was remote from any area of previous periocular radiotherapy.

There is no family history of malignancy, even on interrogation of an extended family pedigree.

This study received ethics approval from Moorfields Eye Hospital Biobank ethics board (15/SW/0104). Written, informed consent was obtained from the family for participation in research and from the patient to publish her clinical history, imaging and genetic results.

DNA was extracted from the orbital tumour by standard methods and analysed for point mutations in common driver genes for cutaneous melanoma (*BRAF*, *NRAS*, *KRAS*) and ocular melanoma (*GNAQ*, *GNA11*); primer sequences and PCR conditions are available upon request. Sequencing of *NRAS* exon 3 showed a heterozygous point mutation (c.A182G; p.Q61R) (Fig. 1e). Sequencing of orbital tumour DNA was also performed for genes related to melanoma prognosis (*SF3B1* and *EIF1AX*), with no somatic mutations being found. MLPA analysis for copy number variation indicated disomy of chromosome 3, with gain of chromosome 6p.

Genomic DNA extracted from peripheral blood under standard conditions was analysed by next-generation sequencing [Hereditary Cancer Syndromes, Comprehensive Diagnostics (ID 93.00); MGZ, Munich, Germany] – this being a multigene diagnostic panel testing for mutations in 94 genes that have been associated with familial-cancer or multiple-cancer syndromes. No germline mutations were shown for this patient.

Our patient is enigmatic on two counts: first, she has survived a perhaps unparalleled period of more than 15 years with cutaneous melanoma metastatic to the orbit, bone, peritoneum and brain. Second, she has had four different primary malignancies, these being cutaneous melanoma, melanoma of soft parts, breast adenocarcinoma and cutaneous squamous cell carcinoma.

Metastatic malignant melanoma has a poor prognosis, with the 5-year survival (with or without residual disease) for stage IV cutaneous melanoma being 15–20%, and about 10% survival by 10 years 1. Tumour metastases to the brain are associated with significant neurological deficit and a very poor survival – less than 8% having a 2-year survival 2 and, without treatment, cerebral metastatic melanoma has a dismal prognosis, with a median survival of 3 months 3. The use of BRAF inhibitors possibly improves the outcome for metastatic melanoma treated with local radiotherapy 4–9, although these retrospective clinical studies are susceptible to selection bias 10.

Remarkably, our patient remains well at 15 years after local resection and radiotherapy (without immunotherapy) for cutaneous melanoma metastatic deep in her left frontal lobe, as well as metastatic deposits in the orbit, femur and retroperitoneum. There are sporadic case reports of patients with long survival following surgical excision of intracerebral metastases, but these are very rare. For example, a case reported in 1950 reports survival of at least 3 years 9 months following the removal of melanoma deposit in the left frontal lobe 11. Two further cases of prolonged survival were reported in 1968, with one patient living for 14 years and a second patient surviving 10 years, both following surgical excision of solitary intracranial metastases 12. More recently, a case reported disease-free survival of 16 years following surgical resection, stereotactic radiation and chemotherapy for multiple intracranial melanoma lesions 13. It is clear, then, that on rare occasions, surgical metastasectomy – with or without adjuvant therapy – can be associated with surprisingly good outcomes. In this case, the patient underwent metastasectomy at three sites (retroperitoneal, intracerebral and orbital). A large analysis of over 4000 patients with metastatic cutaneous melanoma suggested that metastasectomy is beneficial; it was shown that –compared with patients who did not undergo the procedure – patients who underwent metastasis resection had an improved median survival (12 vs. 5 months) as well as higher percentage of 5-year overall survival (16 vs. 7%) 14. It is clear, though, that the case reported here is an outlier to these survival rates and thus metastasectomy cannot be the sole factor that has played a role in her long survival.

It is interesting to speculate whether the identified tumour mutation (*NRAS* p.Q61R) played a role in the long survival in our patient. *NRAS* mutations occur in 13–25% of cutaneous melanoma 15,16, the p.Q61R change accounting for ∼35% of *NRAS*-mutated tumours 17. Advanced cutaneous melanoma containing *NRAS* mutations have a worse prognosis, with shorter median survival and more aggressive disease 18,19. This suggests that our patient’s long survival is not attributable to her tumour driver mutation in *NRAS*. Furthermore, our patient did not have a mutation in *SF3B1* or *EIF1AX*, two mutations associated with a better prognosis in uveal melanoma – although only rarely reported in cutaneous melanoma 20,21. It is, therefore, plausible that our patient’s tumour has a currently unidentified protective mutation and next-generation sequencing of the tumour DNA might be valuable here.

MLPA analysis showed normal disomy 3, but gain of chromosome 6p in our patient’s orbital tumour. Aberrations of chromosome 6 are the most common cytogenetic change in melanomas, with 6p gain in up to 85% of uveal melanomas, and more than 90% of sinonasal and cutaneous melanomas 22. Gain of chromosome 6p (with chromosome 3 disomy) occurs in approximately one-third of uveal melanomas and is associated with better survival 23,24. However, gain of chromosome 6p would normally confer a poorer prognosis in cutaneous melanoma and several other tumours such as colorectal and bladder carcinoma, and sarcoma 22,25. The median survival for patients with melanoma metastatic to the orbit is 24 months after orbital diagnosis, but some patients can have a very long survival (of up to 33 years) 26. However, tumours of cutaneous origin were reported to have the worst survival of all melanoma metastatic to the orbit and thus, again, our patient’s extremely long survival appears to be unusual 26.

The second enigmatic feature of our patient is her multiple primary malignancies, including the particularly rare malignant melanoma of soft parts. With her also developing nonfamilial breast cancer before the age of 50 years, cutaneous melanoma and squamous cell carcinoma, there was a high clinical suspicion that she would carry a germline mutation predisposing to malignancy; notably, however, there was no history suggesting a familial-cancer predisposition syndrome.

The patient’s germline DNA did not show any mutations in 94 known cancer genes, which raises two possible scenarios: first, our patient might just have been ‘unlucky’ and – without any predisposing germline mutation – has developed random somatic mutations giving rise to four separate malignancies. The second possibility is that she harbours an unidentified germline mutation in a very rare oncogene that is not included in the comprehensive diagnostic panel; whole-exome sequencing of her germline DNA might reveal a novel oncogenic or tumour-suppressor gene.

In summary, this patient is a unique case of cutaneous melanoma with multiple metastases, who has survived for a remarkable 14 years after orbital and intracranial disease – despite being treated before the introduction of immunotherapy. Furthermore, she has had four separate primary malignancies – without apparent familial or germline predisposition to cancer.

## References

[1] Ugurel S Röhmel J Ascierto PA Flaherty KT Grob JJ Hauschild A Survival of patients with advanced metastatic melanoma: the impact of novel therapies. Eur J Cancer 2016; 53:125–134. 26707829 10.1016/j.ejca.2015.09.013

[2] Hall WA Djalilian HR Nussbaum ES Cho KH Long-term survival with metastatic cancer to the brain. Med Oncol 2000; 17:279–286. 11114706 10.1007/BF02782192

[3] Fonkem E Uhlmann EJ Floyd SR Mahadevan A Kasper E Eton O Wong ET Melanoma brain metastasis: overview of current management and emerging targeted therapies. Expert Rev Neurother 2012; 12:1207–1215. 23082737 10.1586/ern.12.111

[4] Narayana A Mathew M Tam M Kannan R Madden KM Golfinos JG Vemurafenib and radiation therapy in melanoma brain metastases. J Neurooncol 2013; 113:411–416. 23579338 10.1007/s11060-013-1127-1

[5] Gaudy-Marqueste C Carron R Delsanti C Loundou A Monestier S Archier E On demand Gamma-Knife strategy can be safely combined with BRAF inhibitors for the treatment of melanoma brain metastases. Ann Oncol 2014; 25:2086–2091. 25057167 10.1093/annonc/mdu266

[6] Ahmed KA Freilich JM Sloot S Figura N Gibney GT Weber JS LINAC-based stereotactic radiosurgery to the brain with concurrent vemurafenib for melanoma metastases. J Neurooncol 2015; 122:121–126. 25519302 10.1007/s11060-014-1685-x

[7] Ly D Bagshaw HP Anker CJ Tward JD Grossmann KF Jensen RL Shrieve DC Local control after stereotactic radiosurgery for brain metastases in patients with melanoma with and without BRAF mutation and treatment. J Neurosurg 2015; 123:395–401. 25768829 10.3171/2014.9.JNS141425

[8] Patel KR Chowdhary M Switchenko JM Kudchadkar R Lawson DH Cassidy RJ BRAF inhibitor and stereotactic radiosurgery is associated with an increased risk of radiation necrosis. Melanoma Res 2016; 26:387–394. 27223498 10.1097/CMR.0000000000000268 PMC4943024

[9] Xu Z Lee CC Ramesh A Mueller AC Schlesinger D Cohen-Inbar O BRAF V600E mutation and BRAF kinase inhibitors in conjunction with stereotactic radiosurgery for intracranial melanoma metastases. J Neurosurg 2017; 126:726–734. 27203149 10.3171/2016.2.JNS1633

[10] Chowdhary M Patel KR Danish HH Lawson DH Khan MK BRAF inhibitors and radiotherapy for melanoma brain metastases: potential advantages and disadvantages of combination therapy. Onco Targets Ther 2016; 9:7149–7159. 28003758 10.2147/OTT.S119428 PMC5161425

[11] Reyes V Horrax G Metastatic melanoma of the brain; report of a case with unusually long survival period following surgical removal. Ann Surg 1950; 131:237–242. 15402796 10.1097/00000658-195002000-00010 PMC1616399

[12] McCann WP Weir BK Elvidge AR Long-term survival after removal of metastatic malignant melanoma of the brain. Report of two cases. J Neurosurg 1968; 28:483–487. 5659576 10.3171/jns.1968.28.5.0483

[13] Hamid NA Chandra A Meyer CH Multiple brain metastases from malignant melanoma with long-term survival. Br J Neurosurg 2004; 18:552–555. 15799166 10.1080/02688690400012616

[14] Wasif N Bagaria SP Ray P Morton DL Does metastasectomy improve survival in patients with stage IV melanoma? A cancer registry analysis of outcomes. J Surg Oncol 2011; 104:111–115. 21381040 10.1002/jso.21903 PMC3199373

[15] Ball NJ Yohn JJ Morelli JG Norris DA Golitz LE Hoeffler JP Ras mutations in human melanoma: a marker of malignant progression. J Invest Dermatol 1994; 102:285–290. 8120410 10.1111/1523-1747.ep12371783

[16] Curtin JA Fridlyand J Kageshita T Patel HN Busam KJ Kutzner H Distinct sets of genetic alterations in melanoma. N Engl J Med 2005; 353:2135–2147. 16291983 10.1056/NEJMoa050092

[17] COSMIC. COSMIC, Catalogue of Somatic Mutations In Cancer. Available at: *http://cancer.sanger.ac.uk/cosmic/search?q=NRAS+p.Q61R*. [Accessed 15 January 2017].

[18] Devitt B Liu W Salemi R Wolfe R Kelly J Tzen CY Clinical outcome and pathological features associated with NRAS mutation in cutaneous melanoma. Pigment Cell Melanoma Res 2011; 24:666–672. 21615881 10.1111/j.1755-148X.2011.00873.x

[19] Jakob JA Bassett RL Jr Ng CS Curry JL Joseph RW Alvarado GC NRAS mutation status is an independent prognostic factor in metastatic melanoma. Cancer 2012; 118:4014–4023. 22180178 10.1002/cncr.26724 PMC3310961

[20] Kong Y Krauthammer M Halaban R Rare SF3B1 R625 mutations in cutaneous melanoma. Melanoma Res 2014; 24:332–334. 24709888 10.1097/CMR.0000000000000071 PMC4101881

[21] Kalirai H Coupland SE An update on ocular melanoma. Diagn Histopathol 2015; 21:19–25.

[22] Santos GC Zielenska M Prasad M Squire JA Chromosome 6p amplification and cancer progression. J Clin Pathol 2007; 60:1–7. 16790693 10.1136/jcp.2005.034389 PMC1860600

[23] Aalto Y Eriksson L Seregard S Larsson O Knuutila S Concomitant loss of chromosome 3 and whole arm losses and gains of chromosome 1, 6, or 8 in metastasizing primary uveal melanoma. Invest Ophthalmol Vis Sci 2001; 42:313–317. 11157859

[24] Ehlers JP Worley L Onken MD Harbour JW Integrative genomic analysis of aneuploidy in uveal melanoma. Clin Cancer Res 2008; 14:115–122. 18172260 10.1158/1078-0432.CCR-07-1825

[25] Namiki T Yanagawa S Izumo T Ishikawa M Tachibana T Kawakami Y Genomic alterations in primary cutaneous melanomas detected by metaphase comparative genomic hybridization with laser capture or manual microdissection: 6p gains may predict poor outcome. Cancer Genet Cytogenet 2005; 157:1–11. 15676140 10.1016/j.cancergencyto.2004.06.004

[26] Rose AM Cowen S Jayasena C Verity DH Rose GE Presentation, treatment and prognosis for secondary melanoma within the orbit. Front Oncol 2017; 7:125. 28690979 10.3389/fonc.2017.00125 PMC5481311

